# Bioinspired Adaptive Neuron Enabled by Self‐powered Optoelectronic Memristor and Threshold Switching Memory for Neuromorphic Visual System

**DOI:** 10.1002/advs.202417461

**Published:** 2025-04-07

**Authors:** Yankun Cheng, Junchao Zhang, Ya Lin, Zhongqiang Wang, Xuanyu Shan, Ye Tao, Xiaoning Zhao, Haiyang Xu, Yichun Liu

**Affiliations:** ^1^ Key Laboratory for UV Light‐Emitting Materials and Technology (Northeast Normal University) Ministry of Education 5268 Renmin Street Changchun 130024 P. R. China

**Keywords:** artificial neuron, neuromorphic visual system, self‐powered optoelectronic memristor, visual adaptation

## Abstract

Visual adaptation allows organisms to effectively analyze visual information in varying light conditions by autonomously adjusting photosensitivity, which is essential for the visual system to perform accurate perception in complex environments. In order to realistically implement the functionality of the visual system, the exploration of bioinspired electronics with adaptive capability is highly desired. Herein, a self‐powered optoelectronic memristor based on ZnO/WO_x_ heterojunction is developed, which can exhibit the visual adaptation functions of desensitization and Weber's law. These functions are achieved through the coupling of the photovoltaic effect with electron trapping in the space charge region of the heterojunction. Furthermore, a bioinspired visual adaptive neuron has been constructed, comprising an optoelectronic memristor and a NbO_x_‐based threshold switching memory, capable of directly converting constant light stimuli into dynamic spike trains. Finally, the adaptive image preprocessing is realized, which promotes the improvement of the object recognition accuracy during the overexposed image recognition process. This study offers a novel approach to developing biologically plausible visual adaptation, fostering the future progress of dynamic neuromorphic visual systems.

## Graphical abstract

The self‐powered optoelectronic memristor based on ZnO/WO_x_ heterojunction is developed, exhibiting the visual adaptation functions of desensitization and Weber's law. A bioinspired visual adaptive neuron is constructed, capable of converting light stimuli into dynamic spike trains. The adaptive image preprocessing is realized, which promotes the improvement of the object recognition accuracy during the overexposed image recognition process.

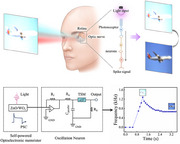

## 1 Introduction

Sensory adaptation is a fundamental characteristic of the sensory nervous system in organisms, which adjusts their internal states in response to environmental alterations.^[^ ^1^, ^2^, ^3^ ^]^ This process facilitates the ascription of more suitable behaviors and activities that are imperative for their survival.^[^ ^4^, ^5^ ^]^ Visual adaptation empowers the human visual system (HVS) to discern a broad spectrum of environmental data with precision, thereby enhancing the capacity to adapt to the ever‐changing world.^[^ ^6^, ^7^, ^8^ ^]^ For instance, the photoreceptors within the retina, consisting of rod and cone cells, are capable of dynamically modulating their photosensitivity, thereby generating an appropriate level of electrical spikes.^[^ ^9^, ^10^, ^11^ ^]^ This ensures the unimpaired operation of the human eye in response to illumination ranging from dim starlight to bright sunlight. There is thus a high demand to develop neuromorphic electronics that emulate the function of visual adaptation, which would promote the advancement of the highly efficient neuromorphic visual system (NVS) facing complex environments.

Previous exploration on visual adaptive devices has primarily focused on constructing circuits using silicon‐based transistors as logic elements, which has encountered substantial challenges like large‐scale integration and high energy consumption.^[^ ^12^, ^13^, ^14^ ^]^ Recent advancements suggest that emerging neuromorphic optoelectronic devices can be considered promising architectures for achieving efficient visual adaptation.^[^ ^15^, ^16^, ^17^, ^18^ ^]^ In particular, adaptive functions have been demonstrated in several relevant material systems using a single device, which typically improved operating efficiency.^[^ ^19^, ^20^, ^21^ ^]^ For example, Liao et al. developed a bilayer MoS_2_ phototransistors that demonstrated visual adaptation through the dynamic modulation of the device photosensitivity under different lighting conditions.^[^ ^22^ ^]^ Chen et al. developed a perovskite photovoltaic sensor to realize adaptive image sensing of the visual information.^[^ ^23^ ^]^ He et al. proposed an organic transistor that incorporates two bulk heterojunctions and is capable of light intensity‐dependent photoadaptation.^[^ ^24^ ^]^ Previous researches has greatly facilitated the development of neuromorphic visual adaptation systems. However, it is still a challenge to realize the characteristics of light‐induced adaptation in a single platform due to the limited materials and physical models. In the HVS, light stimuli with environmental information are encoded by photoreceptors into action potentials (spikes) that are transferred by synapses and neurons.^[^ ^25^, ^26^, ^27^ ^]^ This process enables the brain to learn, adapt, and make decisions with remarkable energy efficiency and very robust to noise.^[^ ^28^, ^29^ ^]^ Therefore, the exploration for neuromorphic devices with adaptive properties that are capable of emulating the real‐time spike encoding function of the retina represents cutting‐edge research.

In this work, we demonstrated an adaptive optoelectronic memristor based on ZnO/WO_x_ heterojunction film. The heterojunction device exhibits transient response and a dynamic adaptation to constant light stimuli at zero bias voltage. The two fundamental visual adaptation functions have been mimicked, including desensitization and Weber's law. The working mechanism for the dynamic adaptation is ascribed to be the synergy of the photovoltaic effect and electron trapping in the space charge region of heterojunction (SCRH). The visual adaptive neuron, comprising an optoelectronic memristor and a threshold switching (TS) memory, exhibits frequency relaxation behavior under constant light stimulation. Moreover, adaptive image pre‐processing and recognition is implemented, possessing high recognition accuracy for overexposed images. The proposed optoelectronic device with biosimilar adaptive encoding capability may be regarded as a building block for advanced neuromorphic visual systems.

## 2 Results and Discussion

**Figure 1:**
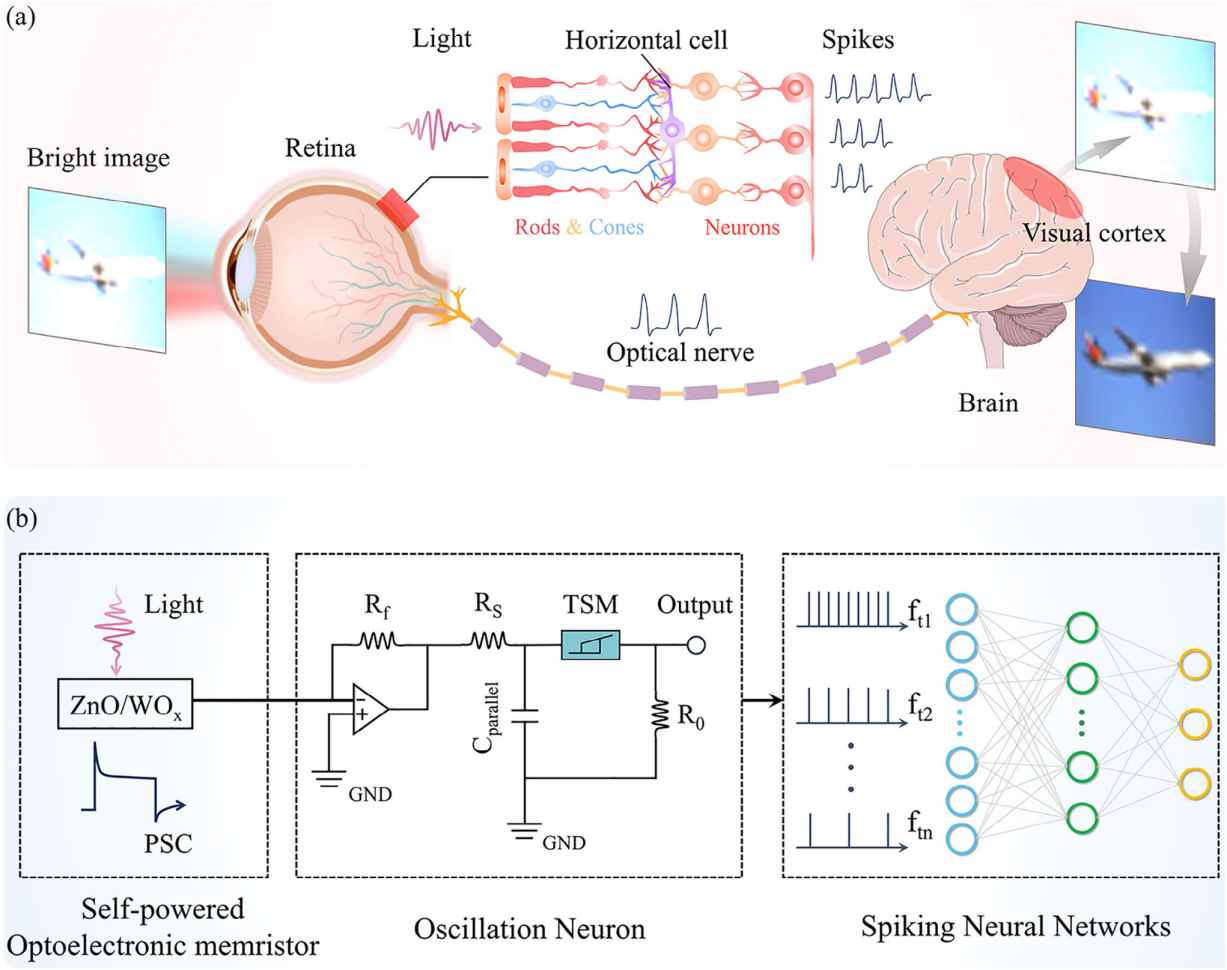
Schematic diagram of the biological and artificial visual systems. a) In the biological visual system, the retina can adapt to varying light conditions through the action of photoreceptor cells, which include cones and rods. The objects under bright illumination scenarios can be well perceived by visual adaptation. Following the retinal adaptation process, the input of an overexposed airplane picture can be converted to a common picture. The adaptation of the retina is achieved by the neuron cells tuning rods and cones, the threshold of the retina, and the frequency of biological spiking to the visual cortex, which are all correspondingly changed. b) Artificial visual systems consisting of a self‐powered optoelectronic memristor, oscillation neuron, and a spiking neural network to emulate visual adaptation. The adaptive optoelectronic memristor is capable of perceiving light stimuli, which generate dynamic postsynaptic currents (PSCs). The oscillation neuron is responsible for converting the output into spike trains. Ultimately, the SNN processes spikes of different frequencies, that carry ambient light information.

**Figure** **1** schematically depicts the motivation of demonstrating visual adaptation functions by employing an adaptive optoelectronic memristor and TS memory for the NVS. The human visual system progressively adapts to the bright light and restores vision over time, which is called visual adaptation, as shown in Figure 1a. The dynamic modulation property of the retina relies on the function of photoreceptors (rod and cone cells) and horizontal cells, which are essential for the visual adaptation.^[^ ^22^ ^]^ The photoreceptors and neurons in the retina can effectively perceive light signals and generate distinct spike patterns in response to different environmental conditions, such as bright or dim lighting.^[^ ^30^ ^]^ The neural spikes, which contain information about the external environment, are transmitted along the optical nerves to the visual cortex for recognition and memory. Inspired by HVS, a neuromorphic visual adaptive system was developed composed of a self‐powered optoelectronic memristor, oscillation neuron, and a spiking neural network (SNN), as shown in Figure 1b. This system is capable of reproducing the dynamic characteristics of biological neurons, holding the potential for highly efficient visual adaptation application.

**Figure 2:**
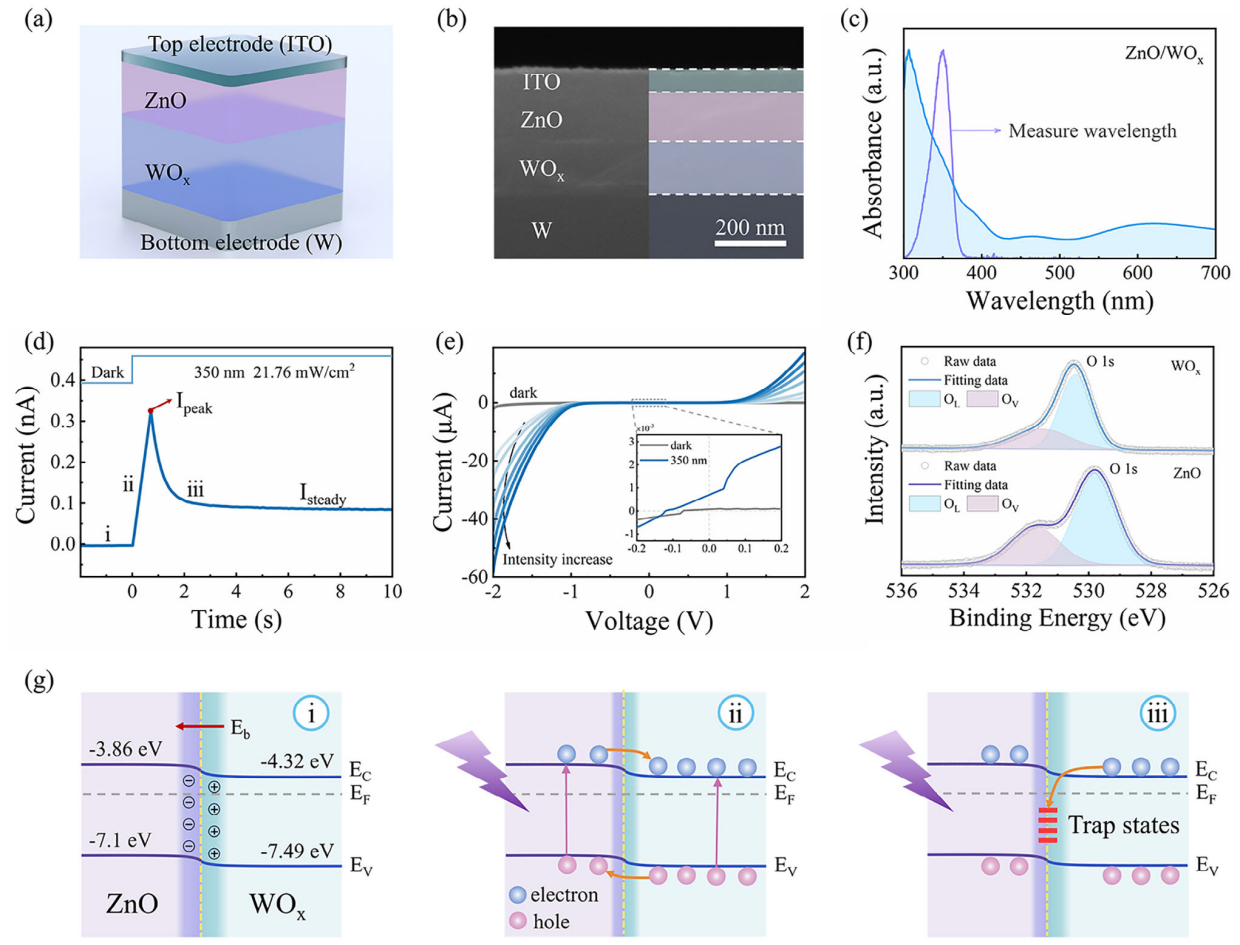
Demonstration of visual adaptive functions and working mechanism of the self‐powered ZnO/WO_x_ heterojunction optoelectronic memristor. a) Schematic diagram of ZnO/WO_x_ heterojunction optoelectronic memristor. b) The cross‐section scanning electron microscope (SEM) image of the device. c) Absorption spectra of the ZnO/WO_x_ film. d) Real‐time photoresponse of ZnO/WO_x_ heterojunction optoelectronic memristor to constant light stimuli on a dark background. e) I–V characteristics of the ZnO/WO_x_ heterojunction device under dark and 350 nm light illumination with different intensities. The inset shows the enlarged I–V curves at the −0.2 – 0.2 V. f) Core level XPS spectra of O 1s from WO_x_ and ZnO films. g) Schematic illustrations of the energy‐band diagrams of ZnO/WO_x_ heterojunction. The working mechanism can be divided into three processes, which correspond to the initial state, the activated process, and the adaptive process, respectively.

**Figure** **2**a,b shows a schematic illustration of our proposed optoelectronic memristor and the corresponding cross‐section scanning electron microscope (SEM) image. The optoelectronic memristor consists of an indium tin oxide (ITO)/ZnO/WO_x_/W structure, fabricated using a sputtering deposition technique. The detailed preparation processes and morphological characterization of the device are presented in the Experimental Section and Figure S1 (Supporting Information). The SEM image of the device that clearly exhibits the multilayer stack with a well‐formed interface between the ZnO and WO_x_ layers. The thicknesses of the ZnO and WO_x_ films are ≈140 and 180 nm, respectively. The temporally‐dependent modulation of ion channel gating in the cellular membrane is regarded as the physiological foundation of the biological adaptive processes^[^ ^31^, ^32^, ^33^ ^]^ (see Figure S2, Supporting Information). The visual adaptive device should possess photoexcitation and dynamic inhibition behavior at the same time. In order to emulate the visual adaptation function of HVS using an optoelectronic memristor, the optical absorption characteristics of ZnO/WO_x_ films were initially assessed. Figure 2c illustrates that the ZnO/WO_x_ film exhibits pronounced UV absorption, validating the choice of 350 nm as the subsequent measure wavelength. Figure 2d demonstrates the photocurrent versus time curves under constant illumination at a wavelength of 350 nm and a light intensity of 21.76 mW cm^−2^. It's worth noting that all the above measurements of photocurrent are conducted under conditions of zero bias, which can verify that the optoelectronic memristive device is self‐powered similar to the previous reports.^[^ ^34^, ^35^, ^36^ ^]^ The results indicate that the response current is composed of three main stages: (i) the initial state preceding the introduction of the light stimulus, which is characterized by a low current; (ii) the current immediately increases to a high current peak (I_peak_) once the light stimulation is applied, exhibiting a discernible current difference and excellent photosensitivity; (iii) the current then reduces gradually to a steady level (I_steady_), demonstrating a desensitization function analogous to that observed in biological receptors. It's worth noting that pure ZnO and WO_x_ films present the increase of current with an absence of inhibition processes under UV light stimulation, indicating that the constant light‐induced adaptation behavior in our device is attributed to its heterojunction structure (see Figure S3, supporting information). Moreover, Figure S4 (Supporting Information) presents the light intensity‐dependent current response of the self‐powered optoelectronic memristor. The light intensity changes from 0.85 to 54.7 mW cm^−2^. Increasing the light intensity resulted in a notable enhancement in both I_peak_ and I_steady_, indicating the dependence of desensitization characteristics on light intensity. In order to further evaluate the recovery property of the optoelectronic memristor, the current response triggered by a pair of light pulses with different time intervals was investigated. As shown in Figure S5 (Supporting Information), the peak current response triggered by the two light pulses with an interval of 2 s decreases from 0.33 to 0.23 nA. Increasing the interval time will decrease the difference of peak current, which becomes equal when the interval is 40 s.

To further understand the working mechanism underlying the self‐powered light‐induced adaptation behavior of the device, the current‐voltage (I–V) characteristics of the optoelectronic memristor were measured and plotted in Figure 2e. These measurements were taken under dark conditions and with 350 nm illumination, varying the intensity from 4.84 to 54.7 mW cm^−2^. The ZnO/WO_x_ optoelectronic memristor exhibited obvious rectification characteristics in both dark and light conditions. The output currents demonstrate a monotonic rise as the light intensity is increased. More importantly, the enlarged I–V curves reveal the photovoltaic characteristics of the device, we can obtain the short circuit currents of 0.7 nA, which endorses the self‐powered capability of the ZnO/WO_x_ heterojunction optoelectronic memristor.^[^ ^37^, ^38^ ^]^ This phenomenon can be attributed to the well‐aligned band structure of the ZnO and WO_x_. As shown in Figure S6 (Supporting Information), Tauc plot analysis^[^ ^39^ ^]^ and X‐ray photoelectron spectrometer (XPS) valence band (VB) spectra^[^ ^40^, ^41^ ^]^ was employed to determine the respective band gaps and valence band maximum (VBM) positions of the WO_x_ and ZnO film materials. The band gaps are ≈3.17 and 3.24 eV, and the VBM is extracted to be 2.85 and 1.83 eV below the Fermi level for WO_x_ and ZnO, respectively. Furthermore, the work functions are calculated using Kelvin probe force Microscope (KPFM) results (see Figure S7, Supporting Information).^[^ ^42^, ^43^ ^]^ The work functions of WO_x_ and ZnO were calculated to be 4.64 and 5.27 eV, respectively, which is consistent with previous reports.^[^ ^44^, ^45^, ^46^, ^47^ ^]^ To evaluate the surface the chemical status and composition of the elements in the films, the XPS analysis was carried out and the results are shown in Figure 2f. The O1s peak of WO_x_/ZnO can be deconvoluted into two components, namely the O_L_ and O_V_ peaks, which are located at 530.4/529.8 and 531.5/531.7 eV, respectively. These components are related to the contributions of lattice oxygen and oxygen vacancies.^[^ ^48^, ^49^ ^]^ It can thus be concluded that the heterojunction formed by the WO_x_ and the ZnO film is characterized by a significant number of oxygen vacancies at the interface.

Based on the above results, the valence (conduction) bands of ZnO and WO_x_ films are respectively found to be –7.1 eV (−3.86 eV) and −7.49 eV (−4.32 eV), indicating that a type‐II heterojunction was formed at the ZnO/WO_x_ heterointerface (see Figure S8, Supporting Information). Correspondingly, the energy band structures of the three stages illustrated in Figure 2c are presented schematically in Figure 2g. Based on the band structures of the two semiconductors and considering the differences in Fermi levels, it can be concluded that when ZnO comes into contact with WO_x_, the electrons will spontaneously migrate from WO_x_ to ZnO until the Fermi level reaches equilibrium. Remarkably, the change in interfacial charge causes the formation of a positively charged electron depletion layer and a negatively charged electron accumulation layer in WO_x_ and ZnO, respectively, and generates a built‐in electric field (E_b_) that is directed from WO_x_ to ZnO at the interface. In stage (i), in the absence of a light stimulus, the device remains in its original state, accompanied by a negligible dark current, which is attributed to the intrinsic heterojunction properties. However, upon illumination with UV light, photo‐generated electrons, and holes are produced in ZnO and WO_x_. These are then separated by E_b_ finally collected by the W and ITO electrodes, respectively. This process forms the photovoltaic effect‐induced peak current for the stage (ii). Subsequently, in stage (iii), partial electrons would be trapped in the SCRH, resulting in a reduction in electron concentration similar to the previous works.^[^ ^19^, ^20^ ^]^ This process corresponds to desensitization behavior in the ZnO/WO_x_ heterojunction device. The current decay can be suppressed by applying a bias voltage (see Figure S9, Supporting Information). The suppression of photogenerated electron trapping under illumination, achieved through the filling of interface trap states by injected electrons, provides experimental evidence for the SRCH trap state‐induced current decay mechanism.^[^ ^50^ ^]^ The three processes of the working mechanisms are in accordance with the three different states of ion channels illustrated in Figure S2 (Supporting Information), which provides compelling evidence that the proposed device is highly suitable for emulating the biological adaptation behaviors.

**Figure 3:**
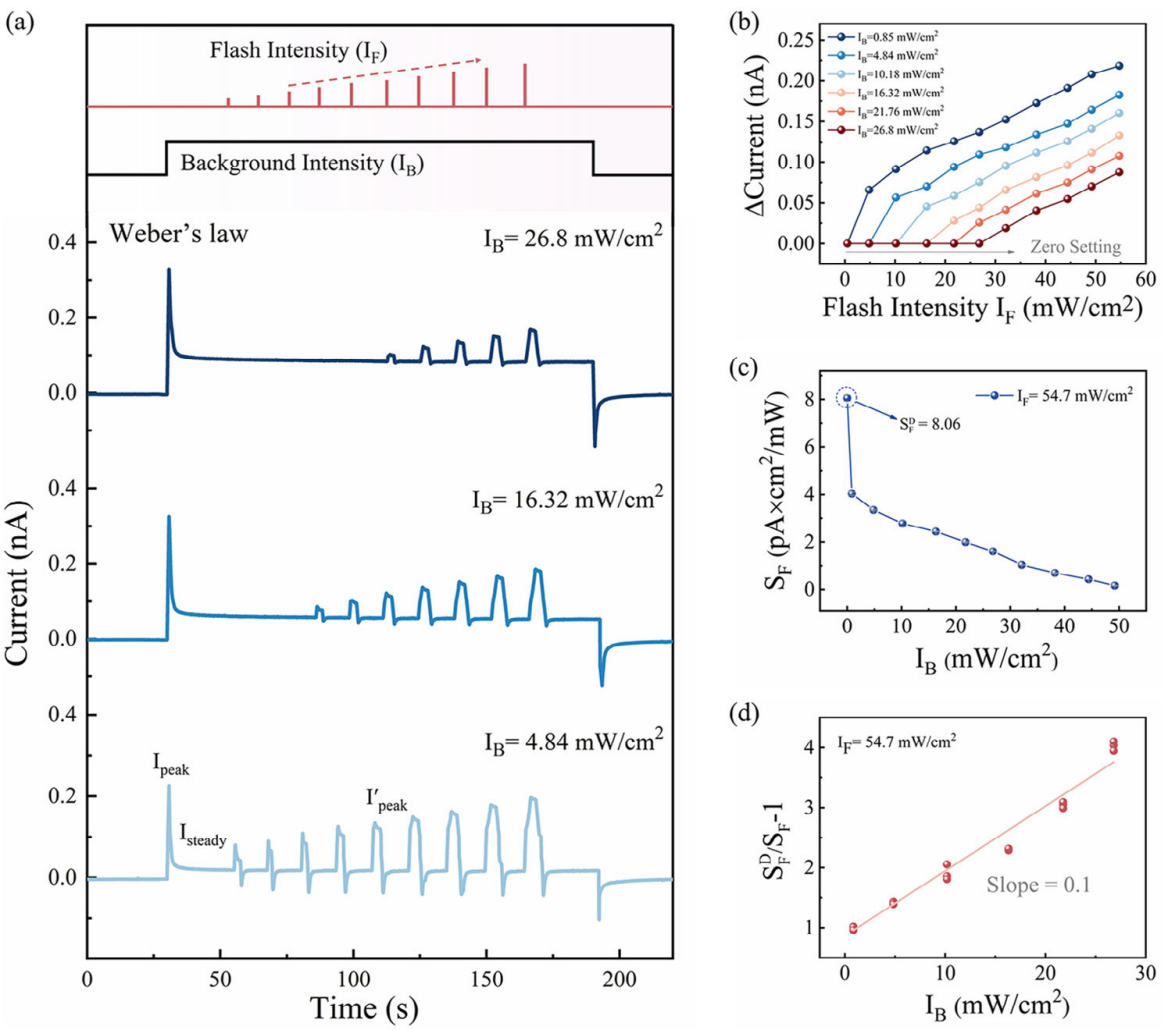
Demonstration of Weber's law functions in ZnO/WO_x_ heterojunction device. a) Photocurrent response of the ZnO/WO_x_ optoelectronic memristor against a background stimulus and flash stimulus. b) The correlation between the amount of current change (ΔCurrent) and flash intensity (I_F_) under different background conditions. c) Correlation between the sensitivity and background intensity. d) The flash sensitivity (S_F_) of the device as a function of background intensity (I_B_).

Weber's law, representing the proportional change in sensitivity with background intensity, is a typical function of sensory adaptation.^[^ ^51^, ^52^, ^53^ ^]^ It enables the organism to identify and distinguish diverse perceptual stimuli from the background. The feature of Weber's law can be reproduced in a single ZnO/WO_x_ optoelectronic memristor, as shown in **Figure** **3**a. To achieve the aforementioned function, the input comprises two distinct types of stimuli. The first is the background stimulus, which is induced by the pulse with a constant intensity (I_B_) and a long duration of 160 s. The second is the flash stimulus, which is induced by the pulse with varying intensity (I_F_) and a short duration of 2 s. The ratio of the duration of the background and flash stimulus is 80, which is comparable to that of biological receptors.^[^ ^52^ ^]^ As shown in Figure 3a, a flash stimulus with I_F_ varying from 4.84 to 54.7 mW cm^−2^ is introduced when the I_B_ induced current reaches a steady state following desensitization. It can be observed that the device in the desensitization state is only responsive to a flash stimulus when the I_F_ is larger than the I_B_. This indicates that the sensitivity of the device to flash stimulus (ΔIʹ = Iʹ_peak_ − I_steady_) is significantly affected by the background condition (I_B_), whereby I_B_ can be conceptualized as the sensitivity threshold. Figure 3b illustrates the correlation between ΔIʹ and I_B_, demonstrating that the zero point of ΔI′ shifts to a higher value with increasing I_B_. This indicates that the sensitivity is reduced by the considerably stronger background stimulus.

In general, the inverse relationship between sensitivity and background intensity is quantitatively described by Weber's law, which is an important psychological law. Weber's law is expressed as follows:^[^ ^52^ ^]^

(1) $$\frac{S_{F}^{D}}{S_{F}}-1=\delta I_{B}$$

where $S_{F}^{D}$ and *S_F_* represent respectively the flash sensitivity of the optoelectronic memristor in conditions of darkness and under background illumination, and δ is a constant. In order to plot the curve of Weber's law, the constants $S_{F}^{D}$ = (I′_peak_ − I_initial_)/I_F_ is calculated according to our experimental results to be 8.06 (Figure 3c). The data represented by S_F_, defined as (I′_peak_ − I_steady_)/I_F_, are extracted from the results presented in Figure 3b. Figure 3d illustrates the curve of Weber's law in accordance with Equation (1), wherein S_F_ is inversely proportional to I_B_ with a slope of 0.1, and the curve bears resemblance to that observed in biological photoreceptors. The capacity of an optoelectronic memristor to alter its sensitivity in accordance with the prevailing background conditions (i.e., Weber's law) enables NVS to adapt effectively to a dynamic environment. Furthermore, the visual perception and photopic adaptation function are demonstrated with fabricated optoelectronic memristor arrays, which benefit from the adaptive feature of desensitization and Weber's law of the ZnO/WO_x_ heterojunction device, as shown in Figure S10 (Supporting Information).

**Figure 4:**
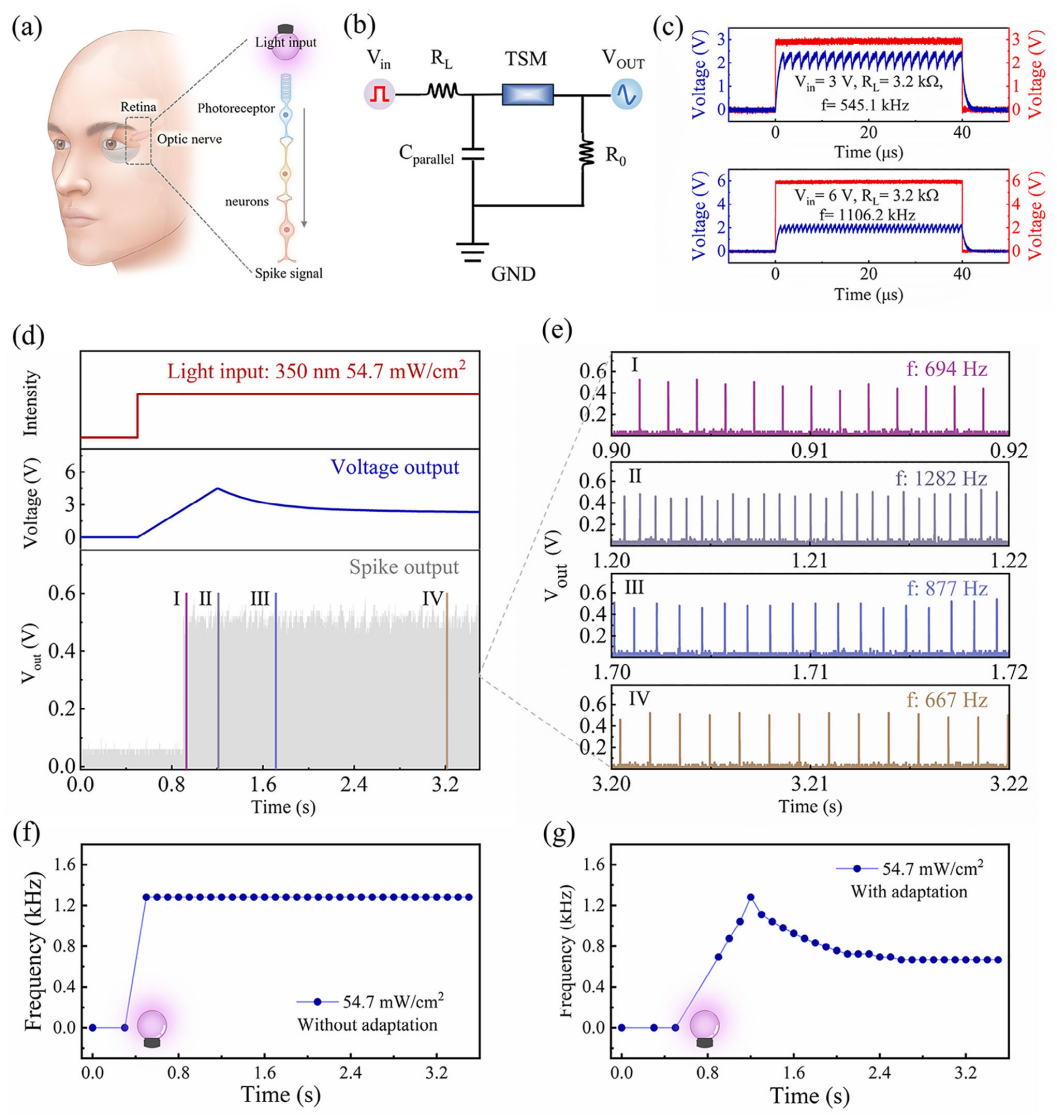
Implementation of the artificial visual adaptive neuron. a) Schematic of concretization of the human visual system. b) Illustration of an oscillation neuron circuit based on NbO_x_ device. c) The oscillation neuron response under different applied voltages. d) The output spikes of visual adaptive neurons in response to constant light stimulation. e) The zoom‐in views of d) at various stages. f) The output frequency of artificial neuron without adaptation as a function of time. g) The output frequency of artificial neuron with adaptation as a function of time.

Humans gather visual information from the external environment and convert it into neural spikes, which are then transmitted to the cerebral cortex for the purposes of learning, memory, and decision‐making (**Figure** **4**a). The NVS with spike coding capability that can efficiently process a variety of visual information is thus desirable. Artificial neurons based on TS memory can generate spikes under external stimuli, which have become ideal building blocks for brain‐like systems.^[^ ^54^, ^55^, ^56^, ^57^ ^]^ The adaptation properties of ZnO/WO_x_ optoelectronic memristor under constant light stimulation, in conjunction with the spike coding capability of the TS memory, provide a foundation for the construction of an artificial visual adaptive neuron that mimics the HVS. The proposed neuron system contains an adaptive optoelectronic memristor, an amplifier circuit and an oscillation neuron, as shown in Figure 1b. The adaptive optoelectronic memristor is responsible for converting a constant light stimulus into a post‐synaptic current (PSC), which is then amplified by the amplifier circuit. The amplifier circuit outputs the desired voltage, which is fed into the oscillation neuron and ultimately generates spike trains.

The TS memory employed in adaptive neuron is based on a NbO_x_ configuration with a vertical meta–insulator–metal structure. The current‐voltage (I–V) characteristics of the NbO_x_ TS memory demonstrate reliable bi‐directional nonpolar volatile resistive switching, as evidenced by 100 cycles (see Figure S11a, Supporting Information). The device switches from the high resistance state (HRS) to the low resistance state (LRS) once the applied voltage surpasses the threshold voltage (V_th_) and immediately reverts to the initial HRS when the applied voltage is reduced below the holding voltage (V_hold_). Furthermore, the threshold switching characteristics of NbO_x_ memory are utilized to construct oscillation neuron, the circuit configuration of which is shown in Figure 4b. Figure 4c shows the spiking patterns of the oscillation neuron under varied input voltage (3V, 6 V) when fixing the R_L_ of 3.2 kΩ and C of 470 pF. The frequency of spikes rises with the increase in input voltage and more results can be found in Figure S11b (Supporting Information). In order to align with the characteristics of the human nervous system (ranging from 1 to 1000 Hz), a 68 nF capacitor was employed to assess spike behavior, resulting in a lower frequency range of 0 to 1300 Hz (see Figure S12, Supporting Information). As shown in Figure 4d, in the presence of a constant light input (350 nm, 54.7 mW cm^−2^), the artificial visual adaptive neuron is observed to encode persistent light into a spike train with a varied level of frequency over time. Figure 4e provides enlarged views of Figure 4d, highlighting the output spikes of visual adaptive neurons at four distinct time periods, labeled as stages I, II, III, and IV. These stages correspond to frequencies of 694, 1282, 877, and 667 Hz, respectively. The visual adaptive neurons are resting in a dark environment. Correspondingly, the frequency of output spikes in adaptive neurons under light exposure demonstrates a time‐dependent dynamic variation. As depicted in Figure 4f, the output spike frequency of the artificial neuron lacking an adaptation function swiftly ascends to 1282 Hz when subjected to intense light exposure (54.7 mW cm^−^^2^), maintaining a constant trigger frequency. Conversely, the output spike frequency of the adaptive visual neuron initially rises gradually to a peak, and subsequently drops over time under the same illumination conditions, as illustrated in Figure 4g. This dynamic modulation of the output spike frequency lays the groundwork for achieving highly efficient image processing. Scuh output frequency of the artificial neuron depends much on the light intensity, where the high intensity can induce a large the peak frequency and long adaptation time, as shown in Figure S13 (supporting information).

**Figure 5:**
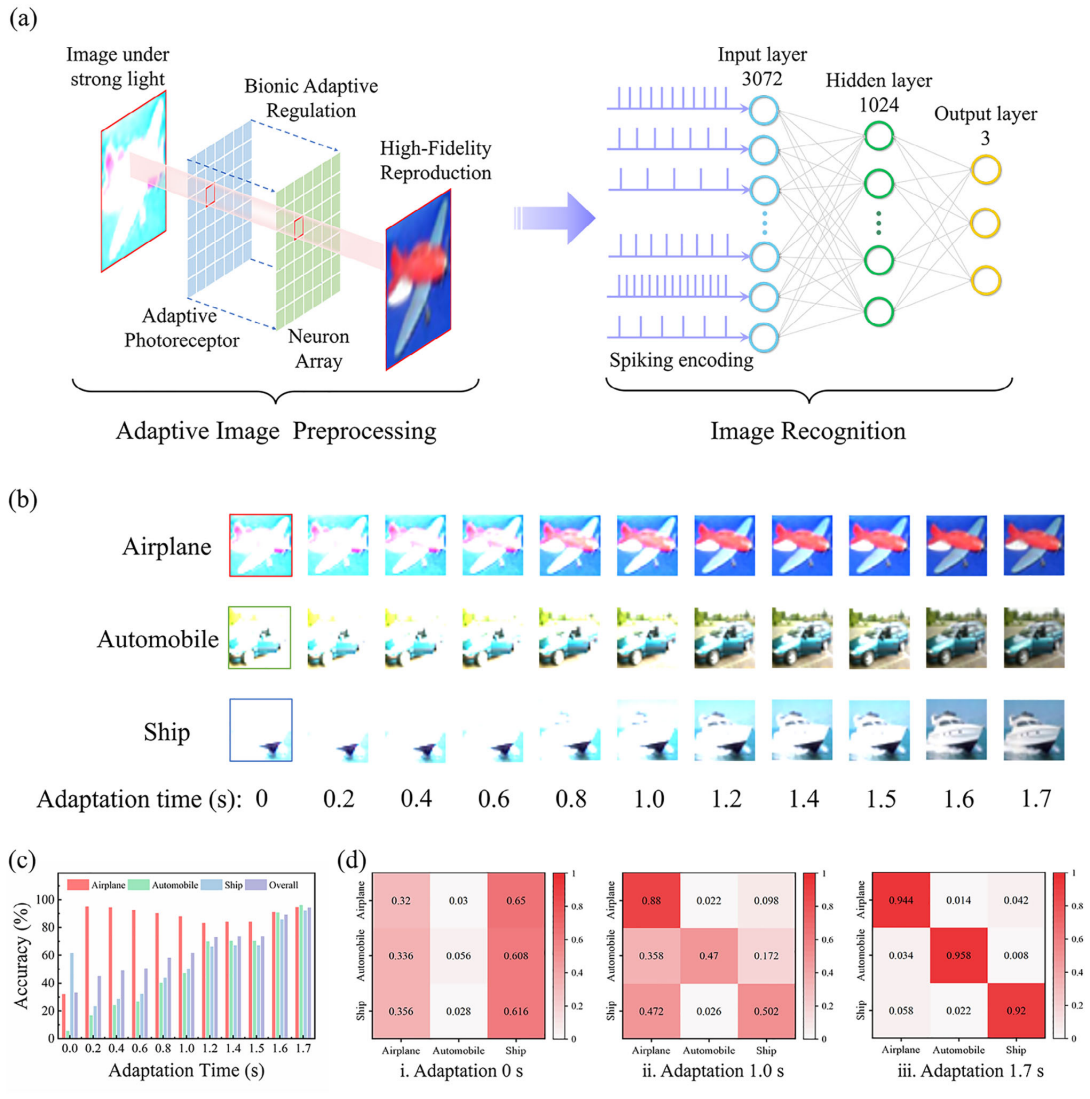
Realization of highly accurate recognition of overexposed images based on visual adaptive neuron. a) Schematic diagram of NVS, which contains the adaptive image preprocessing and image recognition parts based on visual adaptive neuron and SNN, respectively. b) Spontaneous adaption of the overexposed images based on experimental dynamics of the spontaneous relaxation properties in frequency. c) Evolution of the recognition accuracy for images containing airplanes, automobiles, and ships as a function of the adaptation time. d) Evolution of the target object recognition accuracy with the image adaptation time.

The photopic adaptation of the retina plays a crucial role in regulating the activity of post‐neurons in the brain, ensuring that they are not overstimulated by strong light while still enabling us to perceive visual information with optimal clarity.^[^ ^58^, ^59^ ^]^ The spontaneous relaxation of frequency observed in the proposed visual adaptive neuron bears a striking resemblance to the self‐adaptive behavior of image sensing in biological visual systems. Herein, the adaptive image preprocessing was implemented in visual adaptive neuron‐based ZnO/WO_x_ optoelectronic memristors and NbO_x_ TS memory, which enabled the realization of overexposed image recognition with high accuracy. **Figure** **5**a illustrates the schematic diagram of NVS, which encompasses the image preprocessing and image recognition components based on spontaneous relaxation properties in frequency and SNN, respectively. The former is responsible for filtering the overexposed image to remove the background noise and the latter is used to recognize the image. For demonstration, images of airplanes, automobiles, and ships are obtained from the CIFAR dataset of the Keras package and employed for training and recognition tests. Each class of these patterns comprises 5000 samples for training and 500 samples for testing, which are converted into 32 × 32 pixels sub‐images in red (R), green (G), and blue (B) colors. The strength of individual pixel is indicative of the intensity of the reflected light from the object under examination. This can be represented directly in terms of the intensity of the illumination that approaches the optoelectronic memristor during the image production. The preprocessed images are encoded into spikes with different frequencies, which serve as the input to the SNN. The encoded spike trains are then processed by a SNN with three layers which consists of 3072 input neurons, 1024 hidden neurons, and 3 output neurons. The output images can be classified into 3 different categories after training the network, and detailed procedure for the simulation can be found in the Experimental Section.

The relaxation dynamics were extracted from the curve‐fitting of the device frequency by the Boltzmann function, as illustrated in Figure S14 (Supporting Information). Figure 5b demonstrates the feasibility of high‐fidelity reproduction of captured images of airplanes, automobiles, and ships through a stepwise manner by visual adaptive neurons. This facilitates the rectification of the erroneous identification of the targeted object within the images. For example, the untreated overexposed images of airplanes and automobiles will be mistaken as a ship by the SNN, which is due to the formation of featureless white images that lack the requisite target details. The recognition accuracy has been reduced to 32%, 5.6%, and 61.6% for images of airplanes, automobiles, and ships, respectively. An overall accuracy of 33% was obtained, as shown in Figure 5c. With adaptive image preprocessing, although the identification is erroneous at the very beginning, the recognition accuracy rises promptly as the adaptation time increases, and the SNN is capable of correctly recognizing the all three categories of images in 1 s (Figure 5d). Following a 1.7 s adaptation period, a high overall recognition accuracy of 94% was achieved. These results indicate that high‐fidelity imaging and recognition of the examined object in our visual adaptive neuron can promote the development of efficient artificial visual system in complex lighting environments.

## 3 Conclusion

In summary, we have developed a self‐powered optoelectronic memristor based on ZnO/WO_x_ heterojunction to mimic visual adaptation behaviors of biological retina in the HVS. The working mechanism of combining of the photovoltaic effect and electron trapping in SCRH enables the transient response and dynamic adaptation of the optoelectronic memristor to constant light stimulus. The functions of desensitization and Weber's law, as two fundamental cues of visual adaptation, were experimentally demonstrated in a single device. Moreover, an artificial visual adaptive neuron was fabricated by integrating a ZnO/WO_x_ heterojunction optoelectronic memristor and NbO_x_‐based TS memory. The adaptive neuron is capable of mimicking spike‐based visual adaptation behaviors. Furthermore, the overexposed image recognition accuracy can be enhanced' significantly through the implementation of high‐fidelity reproduction by performing the adaptive image preprocessing. Our work provides new insight to develop a highly efficient UV‐ sensitive NVS with adaptive processing capability, which is important for future application of fingerprint recognition and machine vision.

## 4 Experimental Section

### Preparation of the ITO/ZnO/WO_x_/W Device

The W bottom electrodes were deposited on a SiO_2_/Si substrate via radio‐frequency sputtering of the W target at room temperature under a pure Ar atmosphere. The WO_x_ and ZnO films were successively deposited via radio‐frequency sputtering of the WO_3_ (60 W, 30 min) and ZnO (80 W, 30 min) targets at room temperature under Ar/O_2_ with a pressure of 1 Pa. Finally, the ITO top electrodes were deposited on the top of the film via radio‐frequency sputtering of the ITO target at room temperature under a pure Ar atmosphere. The transmittance of ITO top electrodes is above 80%, which was shown in Figure S15 (supporting information).

### Preparation of the Ti/NbO_x_/Pt Device

The Pt/Ti bottom electrodes were deposited on a SiO_2_/Si substrate via radio‐frequency sputtering of the Ti and Pt target at room temperature under a pure Ar atmosphere. The NbO_x_ layer was prepared by radio‐frequency sputtering of 100 W using a Nb_2_O_5_ target at room temperature under a pure Ar atmosphere. The Ti top electrode was prepared by electron‐beam evaporation.

### Electrical Measurements

The optical signals during the measurement were performed with a xenon lamp (LA‐410UV, Hayashi). The device conductance was monitored with the source meter (2636B, Keithley) and probe station (TTPX, Lake Shore). The spike measurements were performed with an arbitrary waveform generator (3390, Keithley; TGA12104, TTi), and a digital storage oscilloscope (DSOS404A, Keysight).

### SNN Simulation

The three‐layer fully connected SNN for the CIFAR dataset recognition was built in Python. The linear LIF neuron in the SNN acted as a ReLU function. The network was trained to minimize the cross‐entropy loss using an Adam optimizer with a learning rate of 0.0005 and a weight decay of 0.0005. The network was trained up to 20 epochs with a batch size of 5.

## Conflict of Interest

The authors declare no conflict of interest.

## Supporting information



Supporting Information

## Data Availability

The data that support the findings of this study are available from the corresponding author upon reasonable request.
