# Supplementary material for: Bioinspired Adaptive Neuron Enabled by Self‐powered Optoelectronic Memristor and Threshold Switching Memory for Neuromorphic Visual System
Source: Adv Sci (Weinh). 2025 Apr 7;12(22):2417461. doi: 10.1002/advs.202417461 (PMC12165122; doi:10.1002/advs.202417461)
Supplement: Supplementary file 1 — Supporting Information [file ADVS-12-2417461-s001.docx]

**Supporting Information**

Bioinspired Adaptive Neuron Enabled by Self-powered Optoelectronic Memristor and Threshold Switching Memory for Neuromorphic Visual System

*Yankun Cheng, Junchao Zhang, Ya Lin*, Zhongqiang Wang*, Xuanyu Shan, Ye Tao, Xiaoning Zhao, Haiyang Xu* and Yichun Liu*

Key Laboratory for UV Light-Emitting Materials and Technology of Ministry of Education, Northeast Normal University, 5268 Renmin Street, Changchun 130024, China

E-mail: liny474@nenu.edu.cn; [wangzq752@nenu.edu.cn](mailto:majg@nenu.edu.cn); [hyxu@nenu.edu.cn](mailto:hyxu@nenu.edu.cn).


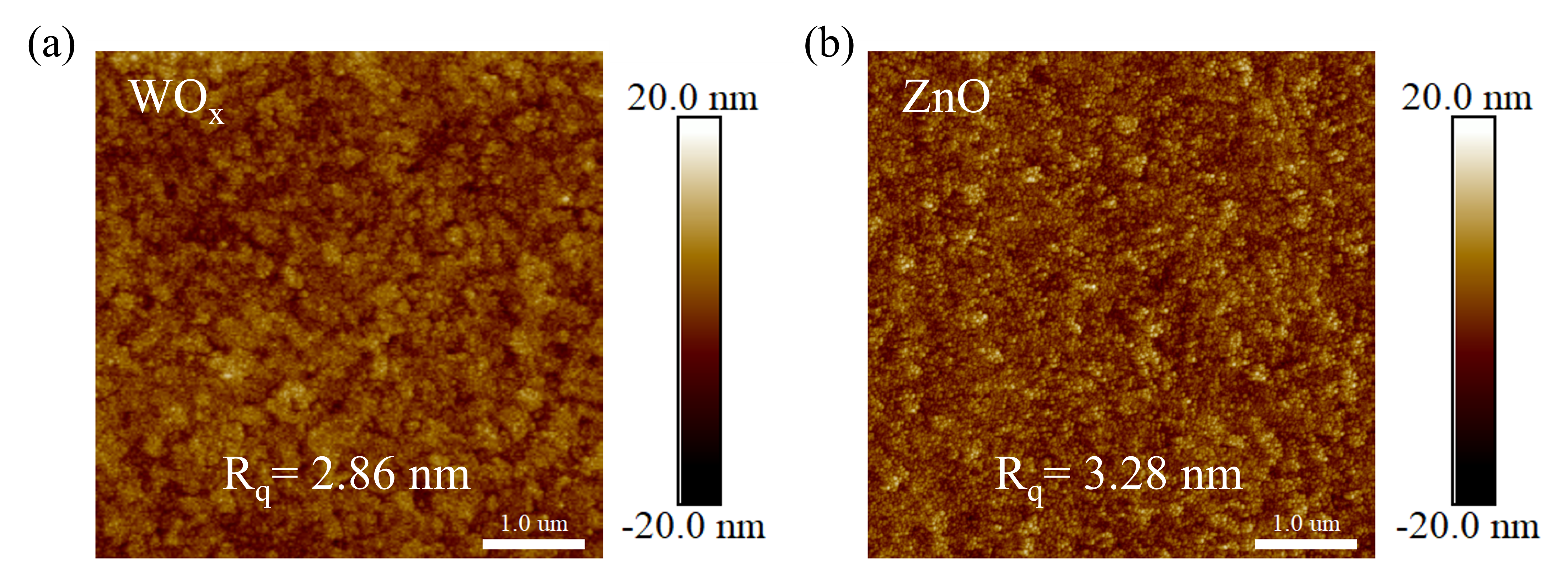


Figure S1. Atomic force microscopy (AFM) height images of (a) WO_x_ and (b) ZnO films. The surface root mean square roughness values (R_q_) of the WO_x_ and ZnO films are 2.86 and 3.28 nm, respectively. These satisfactory levels of smoothness and uniformity ensure the stability of the optoelectronic unit.


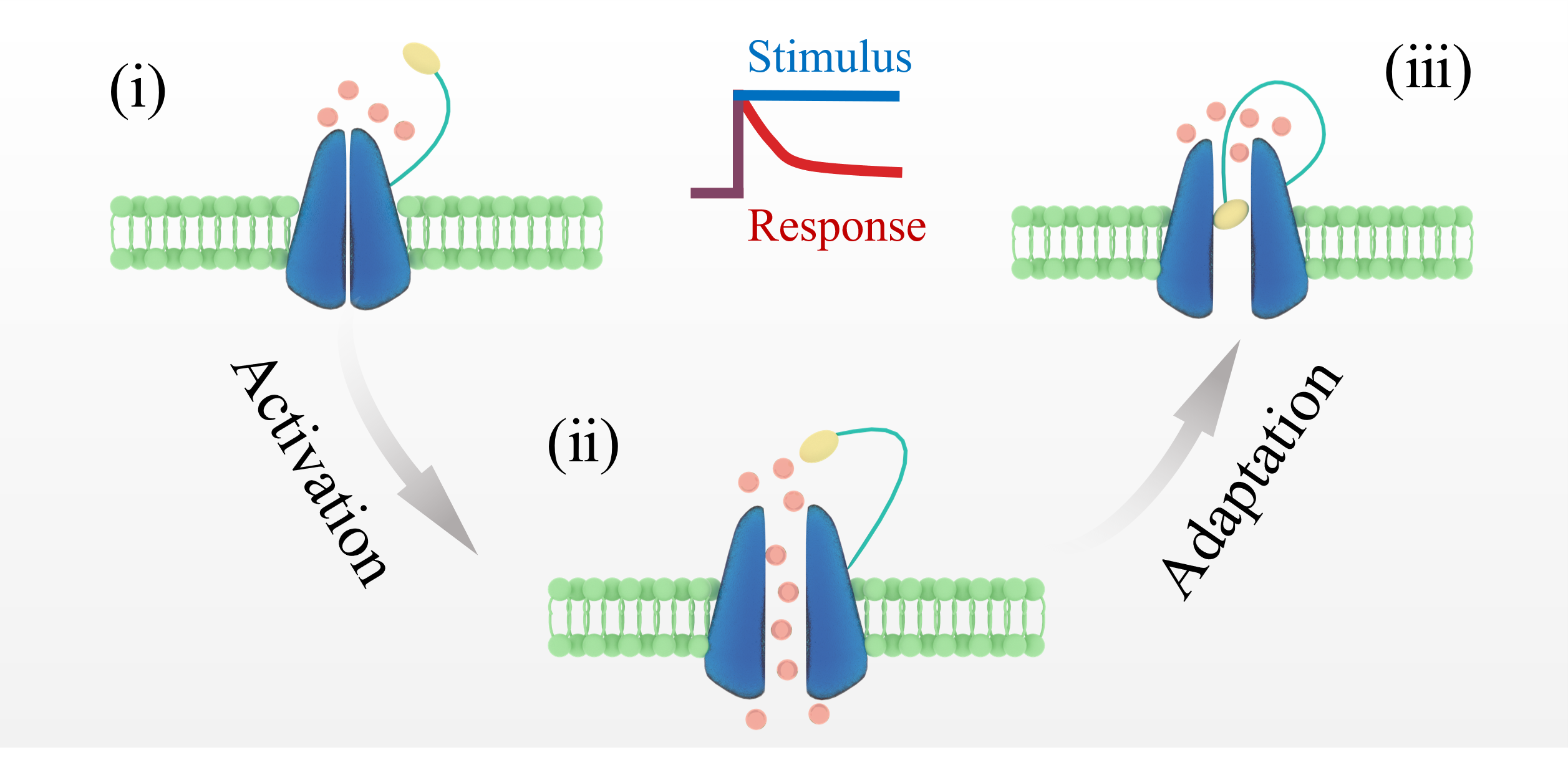


Figure S2. Schematic diagram of adaptation process in biological system. The process involves closure, activation and adaptation of ion channels.

In the absence of stimulus, the ionic channel remains sealed, thereby impeding the conveyance of pertinent signals. Upon the application of an external stimulus, the channel undergoes an exceedingly swift opening, facilitating a surge of ions to inundate the cellular interior, culminating in a peak physiological response. Nevertheless, as the duration of the stimulus persists, the free energy of the channel diminishes, causing its activity to dissipate. This results in a reduction of the ion flux across the cellular membrane, which ultimately leads to progressive adaptation to the external stimulus.


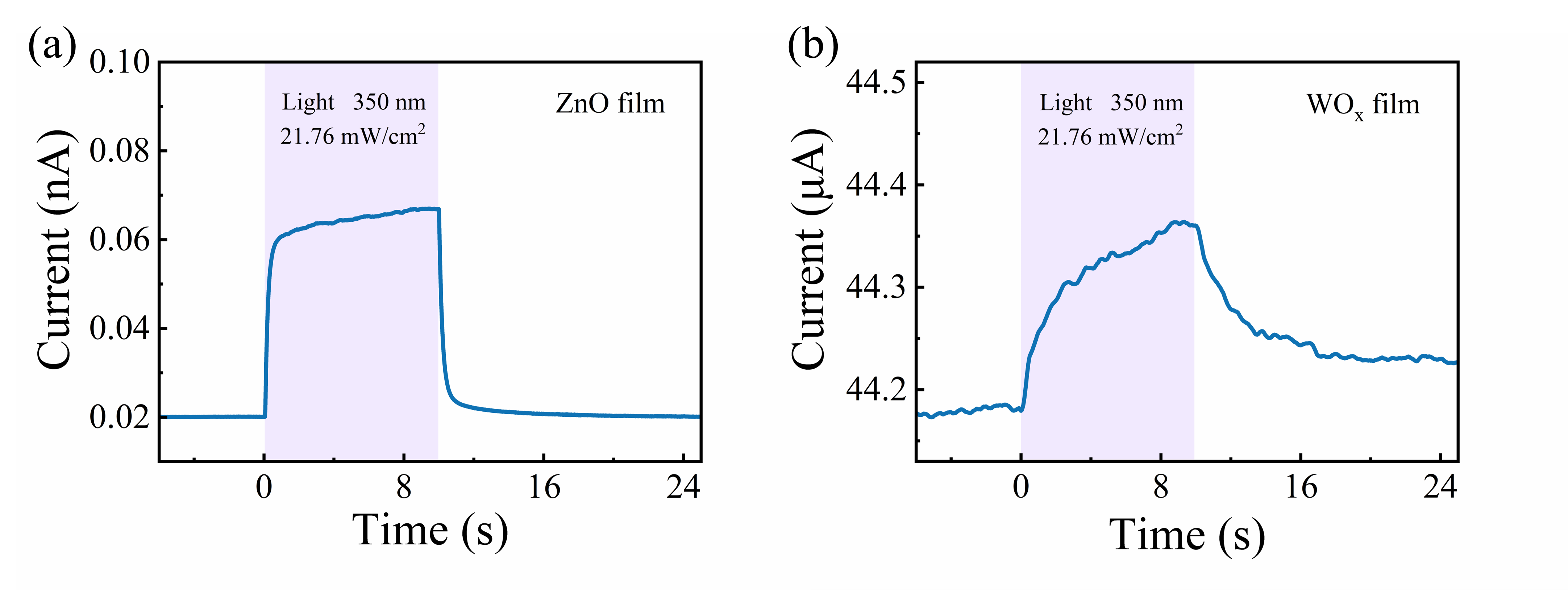


Figure S3. The photoresponse of pure (a) ZnO and (b) WO_x_ film under ultraviolet light illumination (350 nm, 21.76 mW/cm^2^, 10 s).


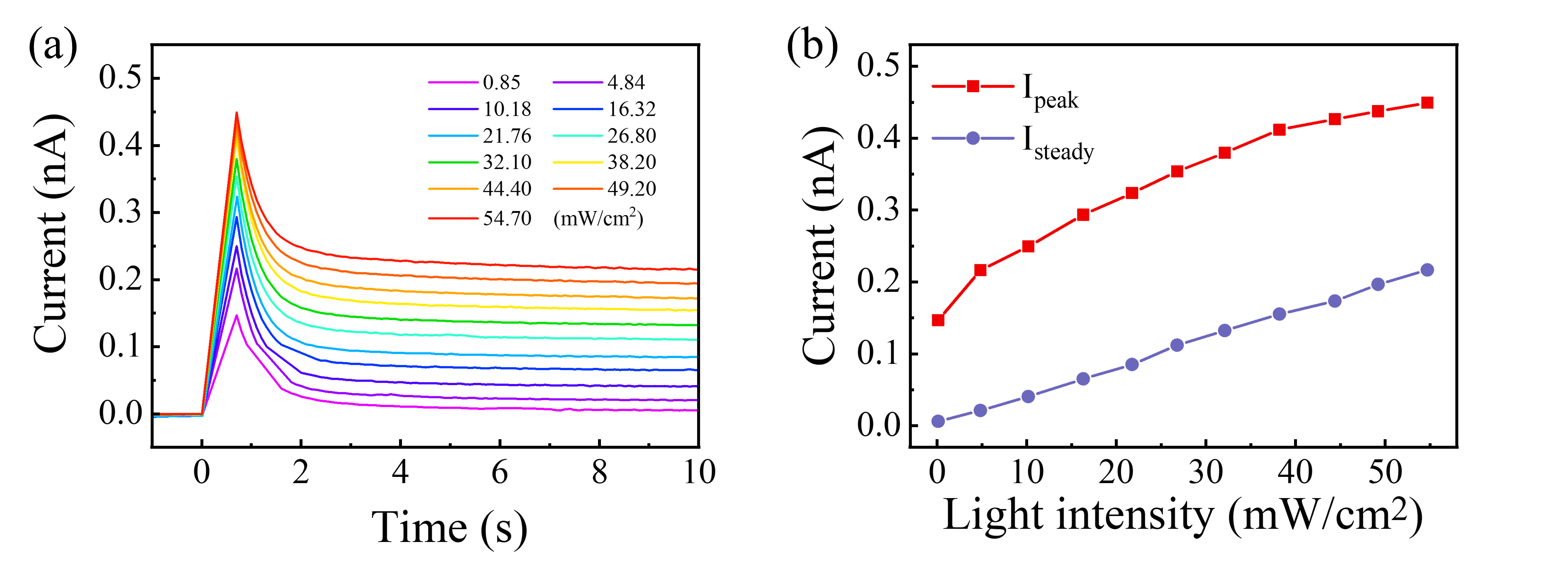


Figure S4. (a) The light intensity-dependent current response of the self-powered ZnO/WO_x_ optoeelctronic memristor. The light intensity changes from 0.85 to 54.7 mW/cm^2^. (b) The dependence of I_peak_ and I_steady_ on light intensity.


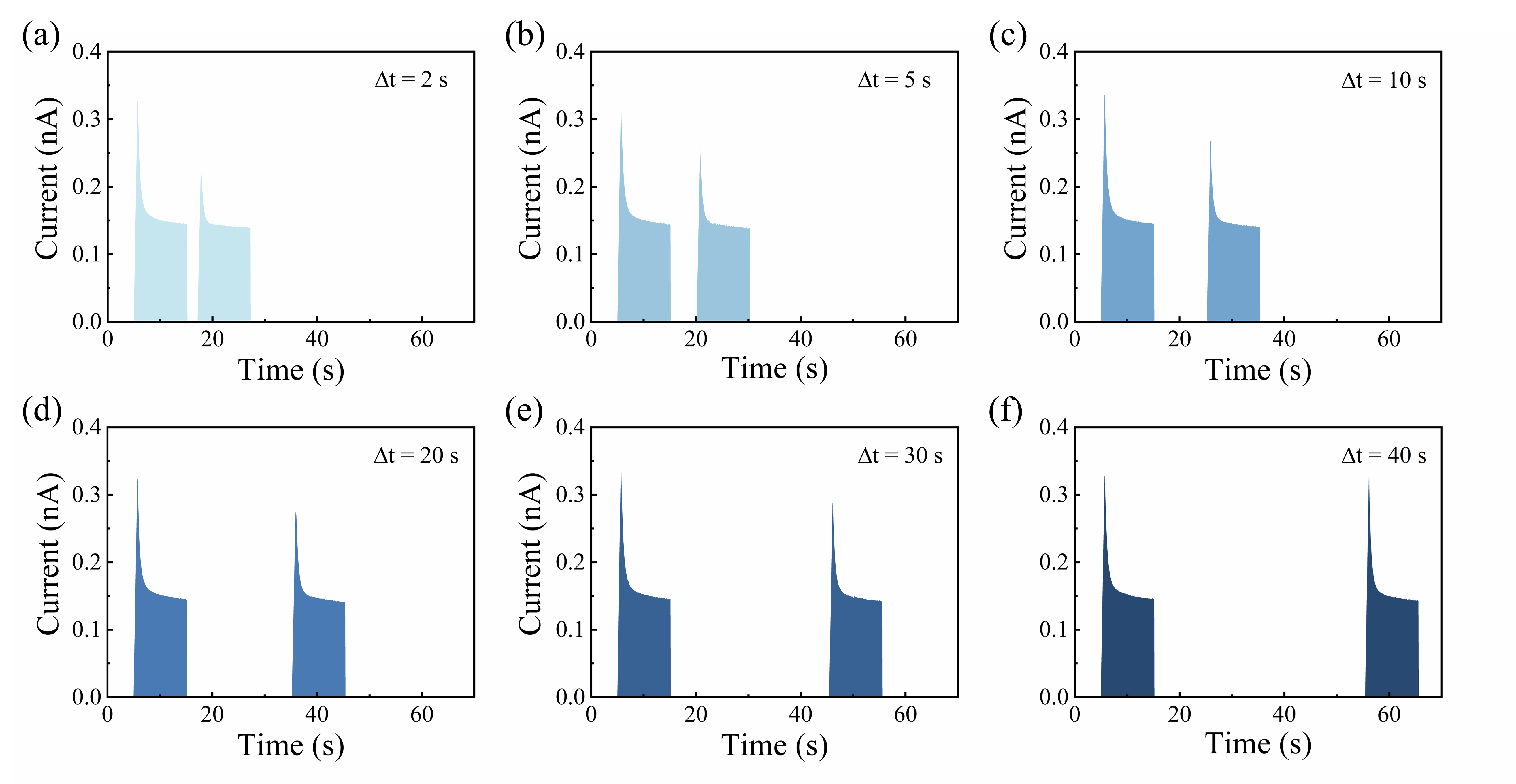


Figure S5. (a-f) Real-time current response to two light pulses with different recovery time intervals. With extension of the time intervals from 2 s to 40 s, the peak current value increases gradually to a stable value.


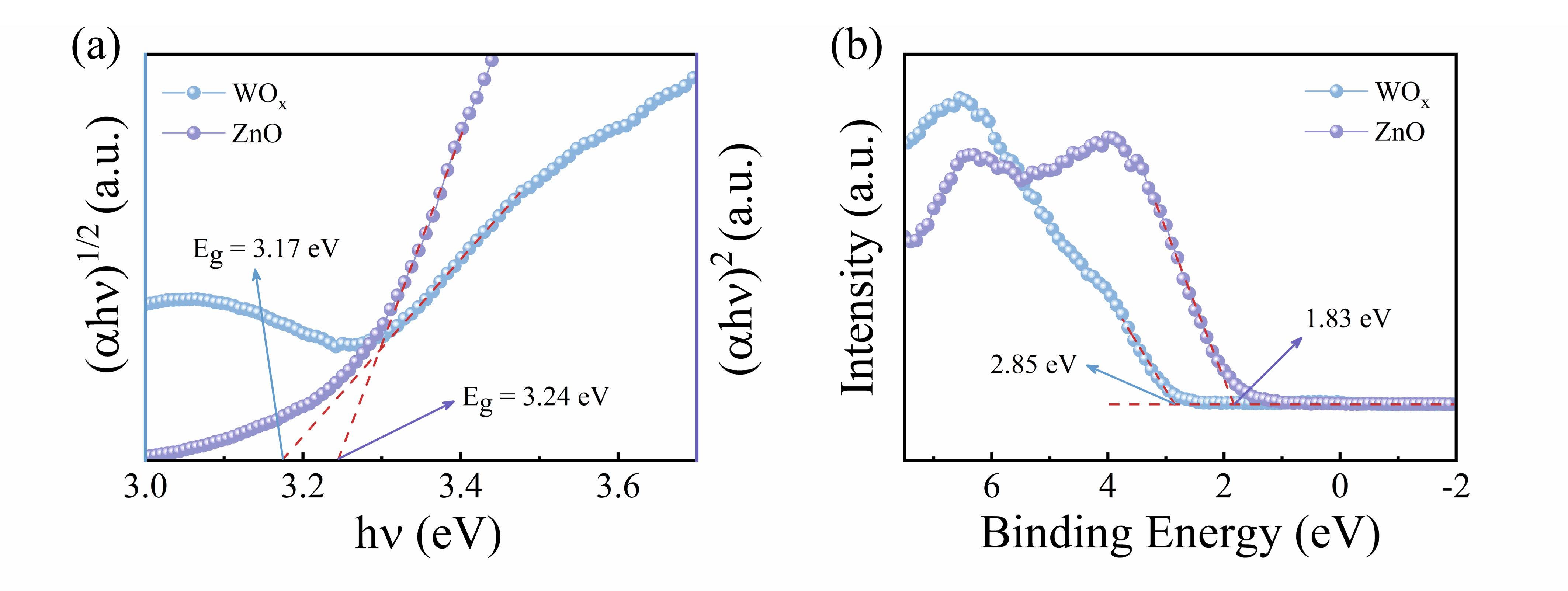


Figure S6. (a) Tauc plots for the extrapolation of bandgaps of WO_x_ and ZnO films. (b) XPS valence band (VB) spectra of WO_x_ and ZnO films, where a linear extrapolation of the leading edge to the extended baseline of the VB spectra is used to determine the valence band maximum (VBM) positions.


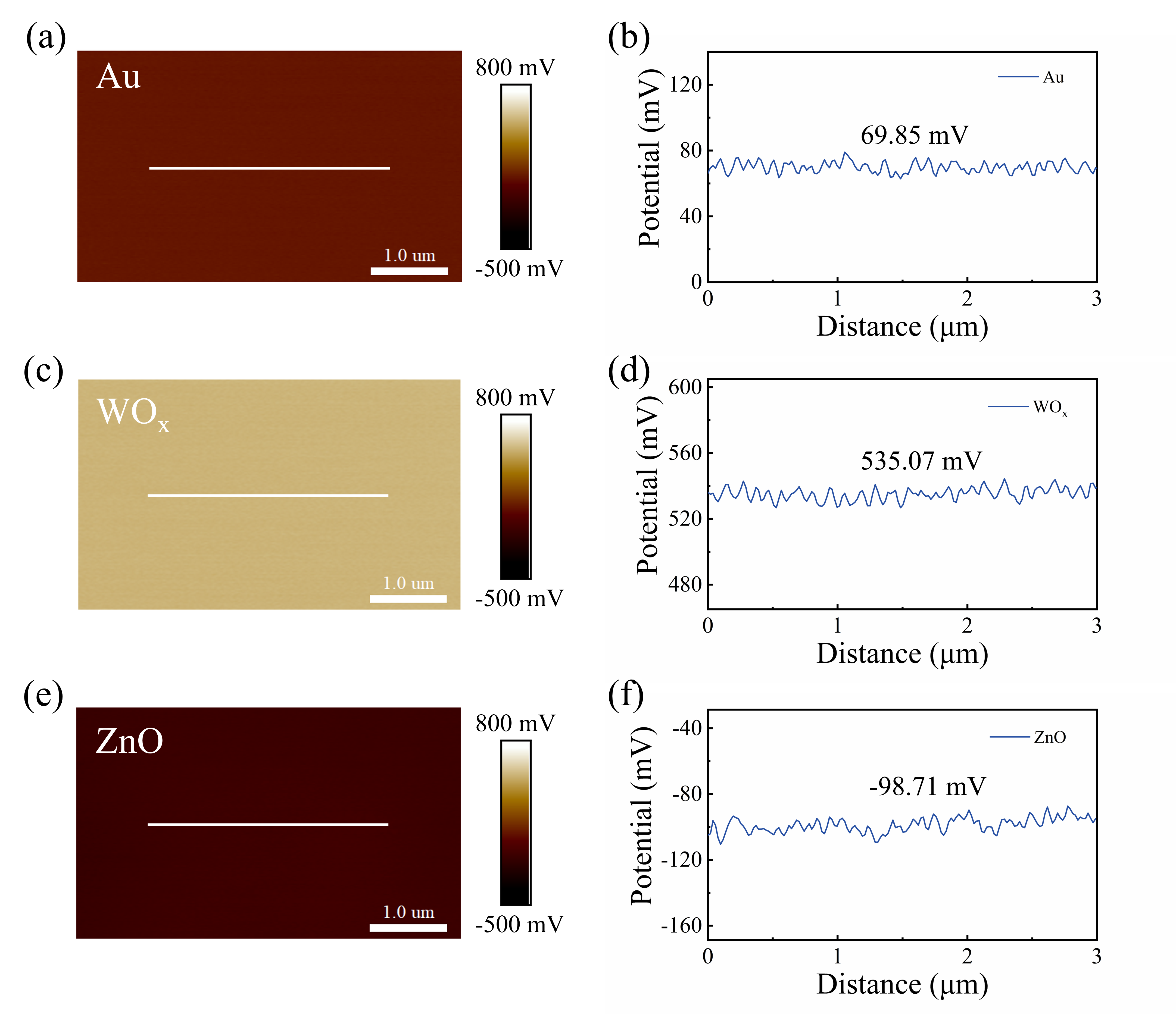


Figure S7. The surface potential images of (a) Au standard sample, (c) WO_x_ film and (e) ZnO film. Corresponding surface CPD values obtained along the white lines of (b) Au standard, (d) WO_x_ film and (f) ZnO film.

The work functions are calculated using Kelvin probe force microscope (KPFM) results, as per the following equation:

$\varphi_{sample}=\varphi_{tip}-V_{CPD}\cdot e$

where $\varphi_{tip}$ is the work function of the KPFM tip, V_CPD_ is the contact potential difference of the KPFM measurements (surface potential), and e is the elementary charge. The average values of the surface potentials of the Au standard sample, WO_x_ and ZnO were obtained from three measurements as 70.35 mV, 534.41 mV and -96.59 mV, respectively. It is known that the work function of the Au standard sample is 5.1 eV, and the equation gives the work function of the tip as 5.17 eV. Consequently, the work functions of WO_x_ and ZnO were calculated to be 4.64 and 5.27 eV, respectively.


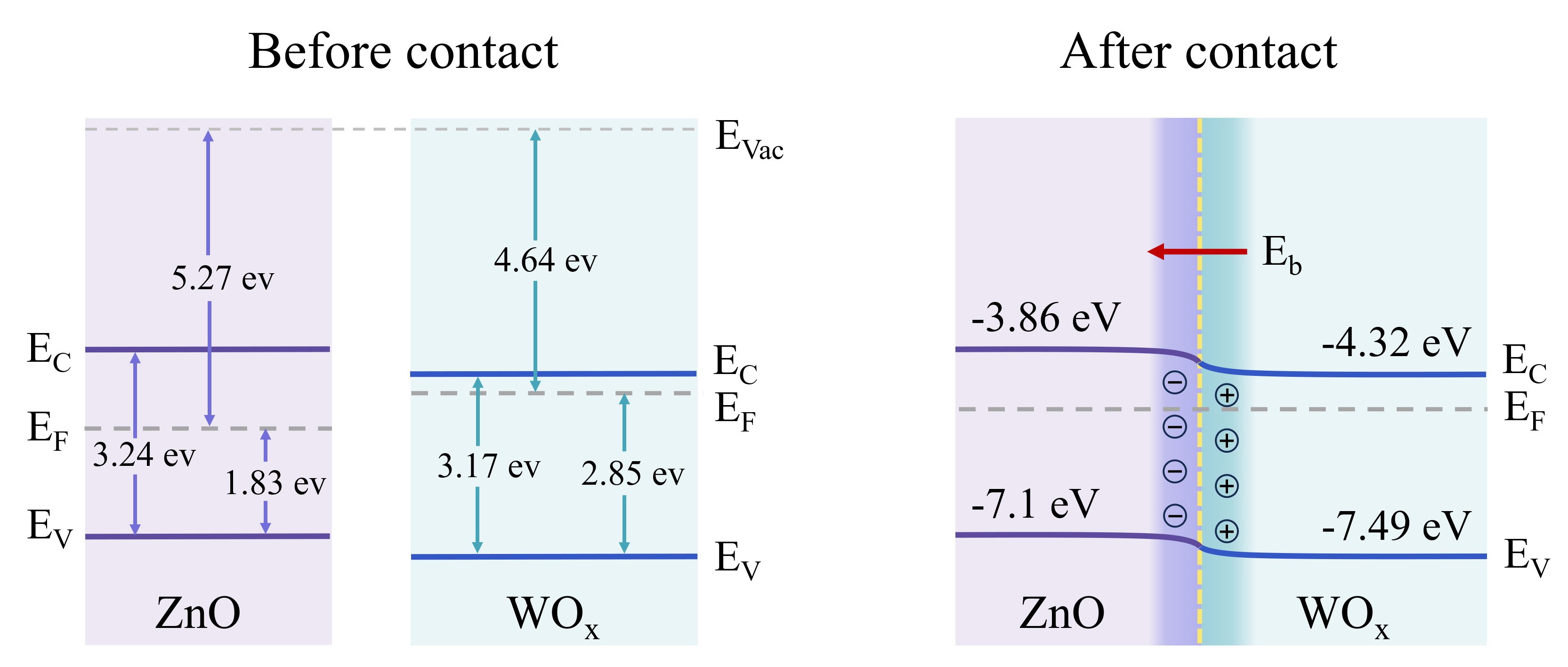


Figure S8. Schematic illustrations of the energy band of ZnO and WO_x_ before and after contact.


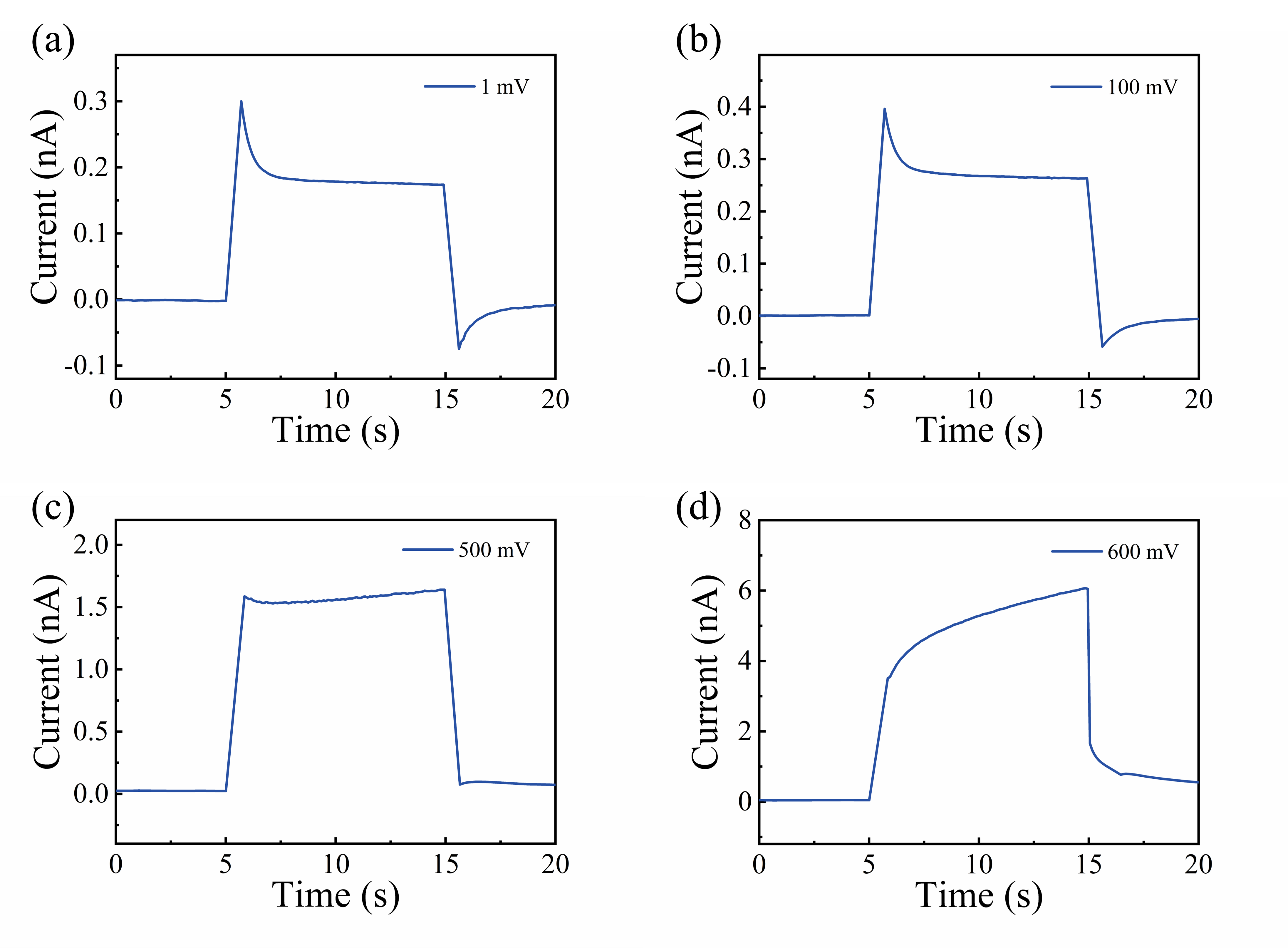


Figure S9. Photocurrent response of the ZnO/WO_x_ heterojunction device across various read voltages, under consistent lighting conditions (26.8 mW/cm^2^, 10 s).


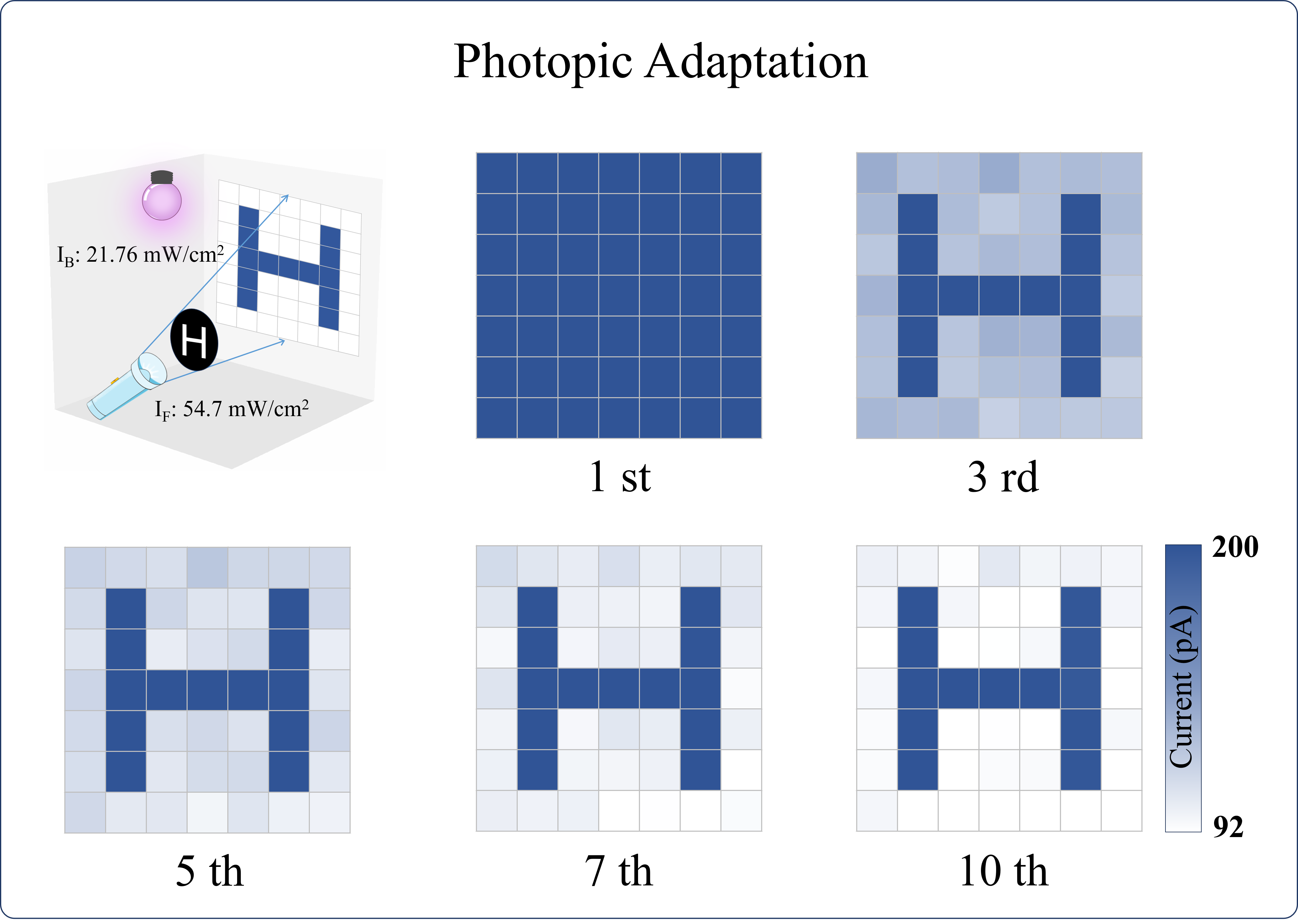


Figure S10. The photopic adaptation process for the “H” pattern with I_F_ of 54.7 mW/cm^2^ and I_B_ of 26.8 mW/cm^2^.

The visual perception and photopic adaptation functions of the human retina are emulated with fabricated optoelectronic memristor arrays, which benefit from the adaptive feature of desensitization and Weber’s law of the self-powered ZnO/WO_x_ device. In order to measure photopic adaptation, a light stimulus of 26.8 mW/cm^2^ is applied to all 7×7 pixels as a bright background, and a light signal with a higher intensity (54.7 mW/cm^2^, 2 s) in a “H” pattern is applied to a subset of the pixels as a flash stimulus. Figure S10 illustrates the perceived evolution of the pattern “H” as the number of flash stimulus applied increases. To quantitatively evaluate the image quality, the image contrast (C) is calculated from the difference in the grey level between the two regions inside the “H” pattern and outside the “H” pattern according to

$C=I_{light}-I_{dark}$

where I_light_ is the average current value of the devices under the flash stimulus and I_dark_ is the average current value of the devices under background stimulus. Herein, the response current ranging from 92 to 200 pA corresponds to the grey level ranging from 0 to 255. Figure S10 shows that the “H” pattern cannot be recognized with zero contrast as a result of saturation of the response current under the bright background in the initial state. Interestingly, “H” patterns are gradually identified from the bright background conditions with the enhancement of the image contrast to 254 as the light pulse number increases to 10, which closely emulates the photopic adaptation in the biological retina. This result is due to the increased sensitivity to flash stimulus caused by the desensitization process. The above results indicate that the ZnO/WO_x_ optoelectronic memristor array exhibits promise for use in image identification under conditions of a changeable background and flash stimulation.


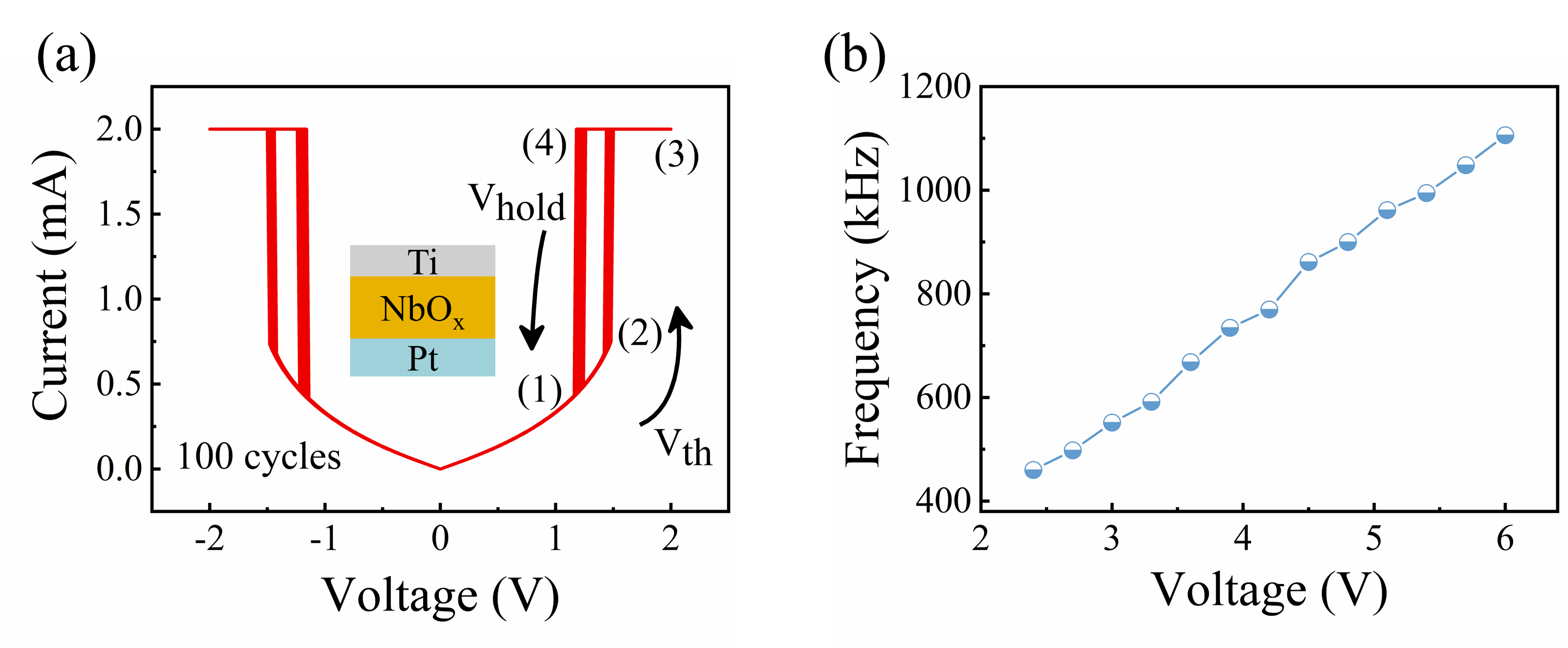


Figure S11. (a) Curren-voltage curves of the Ti/NbO_x_/Pt device in 100 sweep loops. (b) The effect of applied voltage on spiking frequency. The firing frequency increases with the increase of the applied voltage.





Figure S12. The spiking frequency as a function of input voltages. A spiking range from 0 to 1300 Hz matching with the human nervous system (1 ~ 1000 Hz) is obtained.


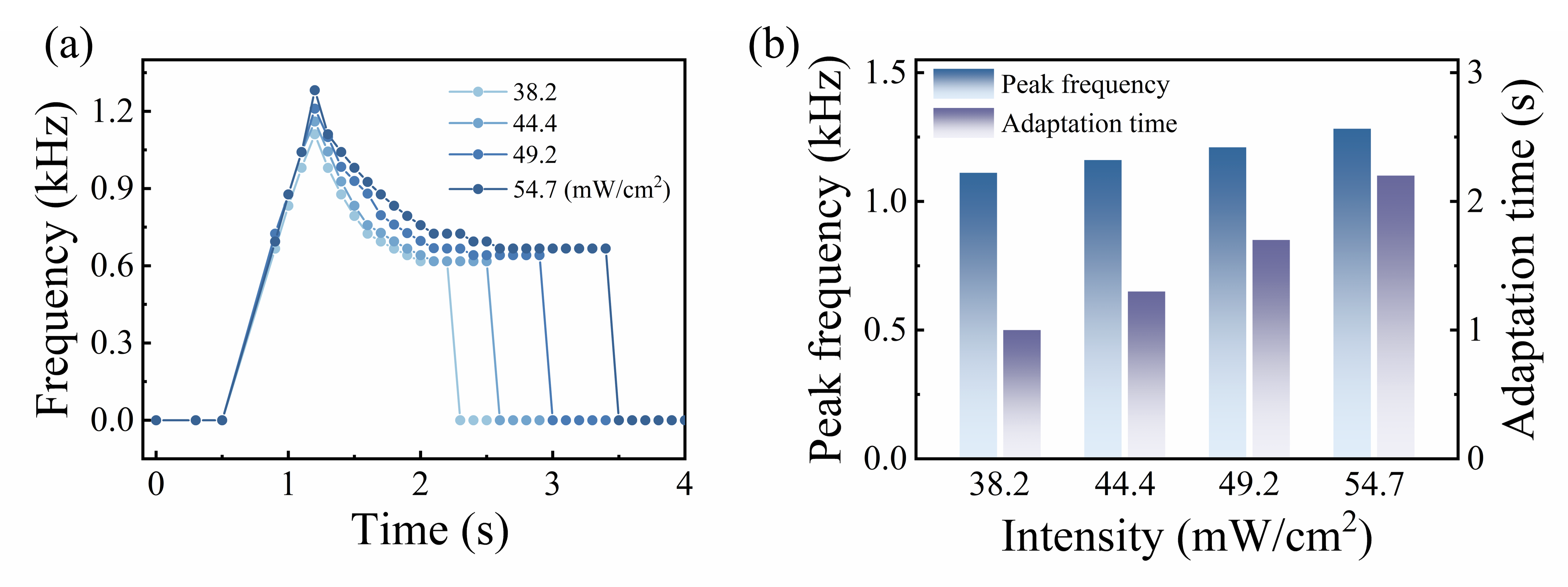


Figure S13. (a) The output frequency of artificial neuron under different light intensities; (b) Statistics of the peak frequency and the adaptation time under different light intensities.


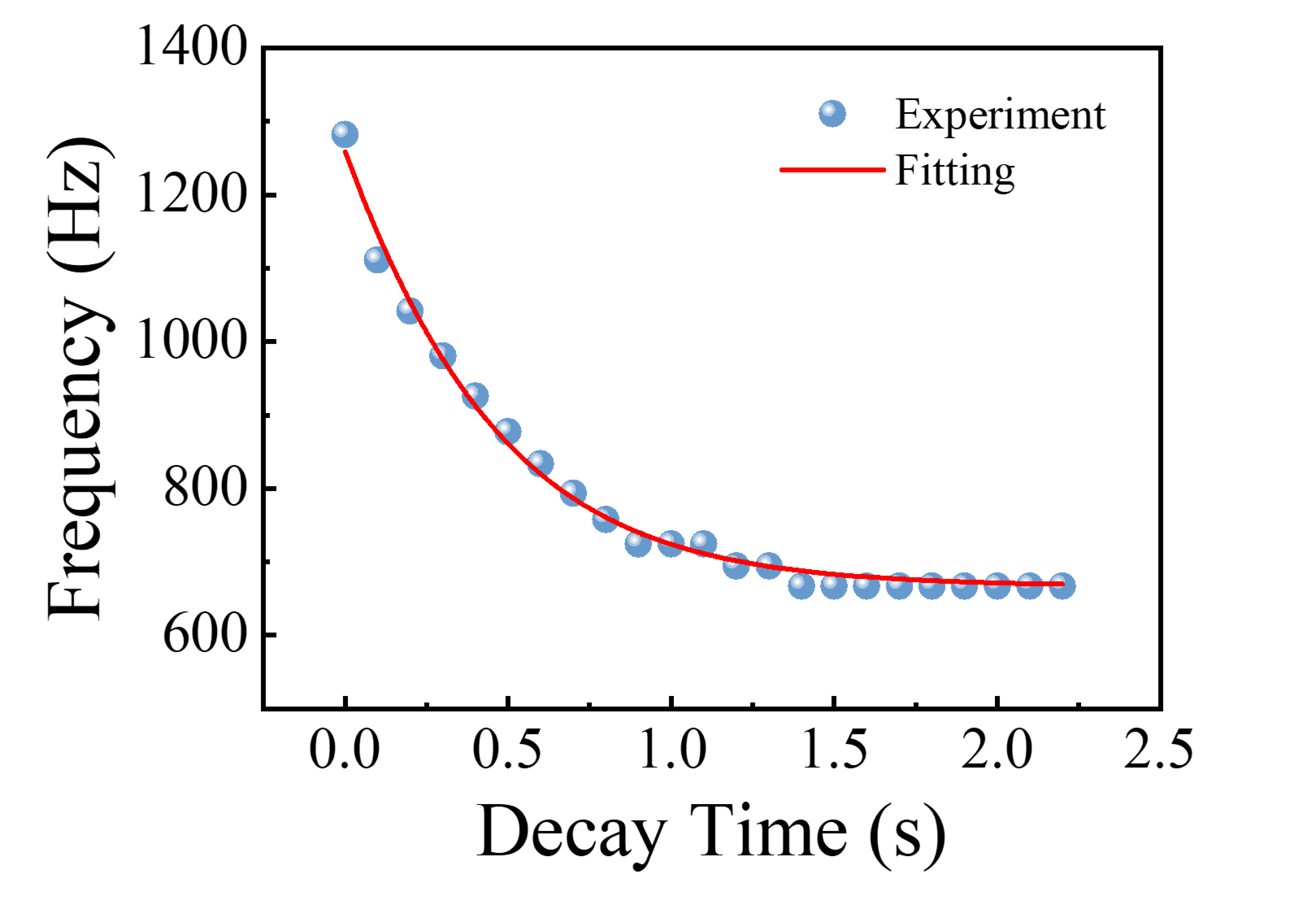


Figure S14. The experimental data and curve fitting of the output frequency in relation to the relaxation dynamics produced by the visual adaptive neuron.

Adaptation of the overexposed images is mathematically conducted according to the relaxation dynamics of the visual adaptive neuron, with the fitted formula:

$$y=x( 667+\frac{2909.8}{1+e^{\frac{t+0.54}{0.39}}} )$$

where y and x represent the RGB weight of the pixel before and after autonomous adjustment and t is the adaptation time. The adjusted weights are used as the input for the SNN.





Figure S15. The transmittance of ITO top electrodes. We can obtain that the transmittance of ITO top electrodes is above 80% at a wavelength of 350 nm.
